# An Interfacial Europium Complex on SiO_2_ Nanoparticles: Reduction-Induced Blue Emission System

**DOI:** 10.1038/srep11714

**Published:** 2015-06-30

**Authors:** Ayumi Ishii, Miki Hasegawa

**Affiliations:** 1 College of Science and Engineering, Aoyama Gakuin University, 5-10-1 Fuchinobe, Chuo-ku, Sagamihara, Kanagawa, 252-5258, Japan

## Abstract

In this study, Eu-coated SiO_2_ nanoparticles have been prepared, consisting of an interfacial complex of Eu and 1,10-phenanthroline (phen) at the solid surfaces of the SiO_2_/Eu nanostructures. The as-prepared SiO_2_/Eu/phen nanoparticles exhibits sharp red emission via energy transfer from the phen to the Eu^III^. After sintering at 200 °C in air, the emission is tuned from red to blue. The blue emission is originated from Eu^II^. This reduction-induced emissive phenomenon resulted from the electron-donating environment created by the surrounding phen and SiO_2_, which is the first reported fabrication of a stable Eu^II^-based emissive material using mild conditions (reaction in air and at low temperature) and an organic-inorganic hybrid nanostructure. The existence of two different stable oxidation states with characteristic emissions, blue emissive Eu^II^ and red emissive Eu^III^, suggests significant potential applications as novel luminescent materials with inorganic-organic hybrid structures.

Interfacial nanostructures formed by organic and inorganic materials have great potential to exhibit novel properties not displayed by the original components. The suitable design of such interfaces is the key to fabricating functional organic-inorganic hybrid materials for applications in photonics^1^,^2^,^3^,^4^, electronics^5^,^6^,^7^, magnetic devices^8^,^9^ and catalyses^10^,^11^. Our current research is focused on constructing functional interfaces through the complexation of organic and inorganic materials, with the aim of stabilizing and tuning the photo-physical properties associated with luminescent materials, such as lanthanide compounds.

Here we demonstrate the development of a novel reduction-induced emission system by forming interfacial europium (Eu) complexes on SiO_2_ nanoparticles. The variations in the luminescence properties of Eu ions with changes in their valence state have been widely investigated in many host materials, and it is known that divalent and trivalent Eu ions luminesce in the blue and red spectral regions, respectively. The divalent Eu ion (Eu^II^) shows a broad emission band assigned to the allowed 4f^6^5d → 4f^7^ electric dipole transition, a phenomenon that has been reported in inorganic host materials such as sulfates, phosphates, borates, silicates and aluminates^12^. The trivalent Eu ion (Eu^III^) shows some narrow emission bands assigned to the electric dipole forbidden (Laporte forbidden) transition of the inner-shell 4f orbitals. The existence of two different oxidation states with characteristic emissions as well as the high emission efficiency of Eu^II^ and the high colour purity of Eu^III^ is predicted to allow the fabrication of novel luminescent materials for a wide range of applications, in the event that a process is found that allows the desired species to be readily selected.

There are no natural sources containing Eu^II^. The emission of Eu^II^ is of significantly higher intensity than that of Eu^III^ in any inorganic host material. Thus, in order to prepare luminescent materials containing Eu^II^, it is necessary to reduce Eu^III^ to Eu^II^ in an appropriate matrix, using a reducing atmosphere at temperatures above 1000 °C^13^,^14^,^15^,^16^,^17^,^18^. Although some reports show that this reduction occurs even in air, high temperatures are still necessary, along with a rigid inorganic crystal structure as a host^19^,^20^,^21^. In contrast, Eu^III^ emission can be easily and efficiently obtained by complexation with organic compounds. In such cases, an organic compound having a high absorption coefficient transfers its photo-excitation energy to the Eu ions. The organic compound in the complex not only plays an important role as an energy donor for the Eu ions, but is also able to control the structures and arrangements of the emissive substances at a molecular level through coordination bonds. It is thus expected that a combination of an inorganic matrix with organic compounds will produce novel luminescent materials in which the performance of the Eu ion is enhanced.

In the present study, we have prepared SiO_2_ nanoparticles coated with Eu ions and discovered that an interfacial complex of Eu and 1,10-phenanthroline (phen) forms at the solid surfaces of the SiO_2_/Eu nanostructures, as illustrated in Fig. 1. SiO_2_ nanoparticles were chosen as host materials because of their high availability and thermal stability. This hybrid nanostructure at the interface between inorganic and organic compounds induced significant visible light emission from two different stable oxidation states of the Eu ions.

## Results And Discussion

To coat the SiO_2_ surfaces with Eu ions, SiO_2_ nanoparticles (20 ~ 50 nm) were immersed in a 50 mM ethanol solution of EuCl_3_ at 70 °C for 30 min, resulting in coating of Eu ions on the SiO_2_ surfaces (this material hereafter referred to as SiO_2_/Eu). This colloidal suspension containing the SiO_2_ and Eu ion was subsequently dropped onto a quartz substrate that was then dried at 110 °C for 15 min. In previous reports, interfacial complexation with anthraquinone or cyclopentadiene has been demonstrated at the solid surface of TiO_2_, and has been shown to function as an excellent visible light absorber for photoelectric conversion^22^,^23^. Accordingly, the SiO_2_/Eu nanoparticles were immersed in an ethanol solution of phen at 75 °C for 60 min to form a new material (SiO_2_/Eu/phen). The phen molecule is known to coordinate to Eu ions in a bidentate fashion through bonding of two nitrogen atoms^24^,^25^. In the present work, phen was also assumed to form an interfacial complex with Eu ions at the solid surfaces of the SiO_2_/Eu nanostructures. X-ray photoelectron spectroscopy (XPS) measurements demonstrated the formation of coordination bonds between phen molecules and Eu ions on the SiO_2_/Eu nanoparticles. In the resulting data, the N1s XPS band of phen at 396.7 eV was shifted to 399.7 eV on the higher energy side, indicating that the phen coordinated to the Eu ions on the SiO_2_ nanoparticles (Fig. S1)^26^. The phen molecule never coordinates the surface of SiO_2_ nanoparticles without Eu ions. It suggests that Eu ions exist on the surface of SiO_2_ nanoparticle and form the complexes at the interface between SiO_2_ nanoparticles and phen ligands.

**Figure 2:**
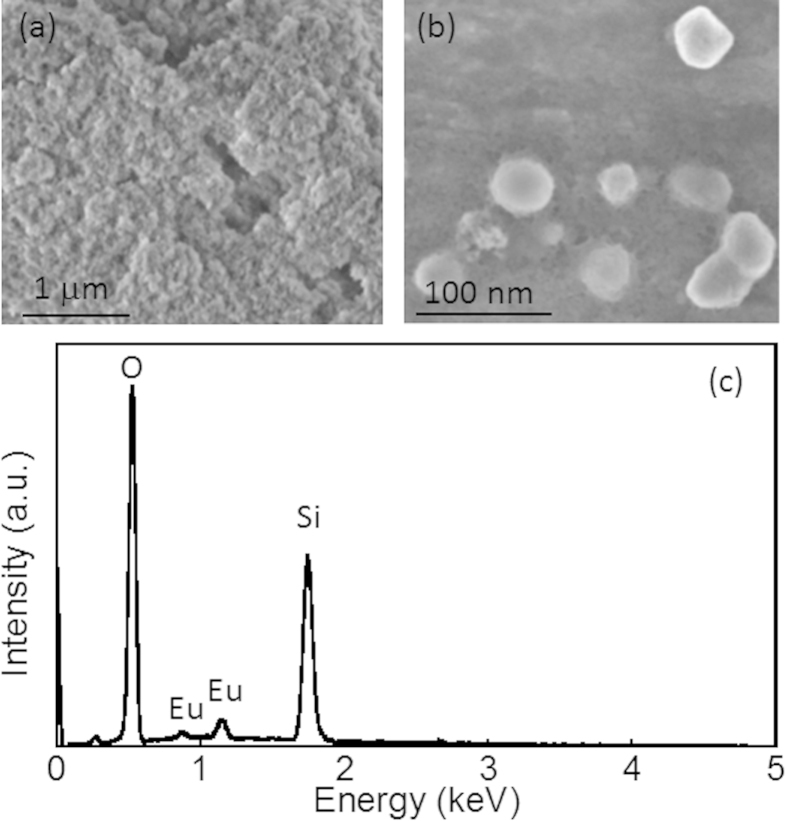
**a**) Low and **b**) high magnification SEM images of SiO_2_/Eu nanoparticles and **c**) the EDS pattern obtained from SiO_2_/Eu nanoparticles.

Fig. 2 shows scanning electron microscope (SEM) images of SiO_2_ nanoparticles coated with Eu ions. Compared with SiO_2_ nanoparticles (Fig. S2), the SiO_2_/Eu particles exhibit close packing between themselves. The average SiO_2_ particle size of 20 nm was almost unchanged by the addition of the Eu ions to the surface, indicating that the ions formed a nano-ordered thin film layer. Energy dispersive X-ray spectroscopy (EDS) was used to determine the elemental compositions, as shown in Fig. 2c. The EDS spectrum confirmed the presence of Si, O, and Eu. Since no Cl peaks were evident around 2.6 keV, we may conclude that Eu oxides or hydroxides were formed on the SiO_2_ nanoparticles through the colloidal suspension process.

**Figure 3:**
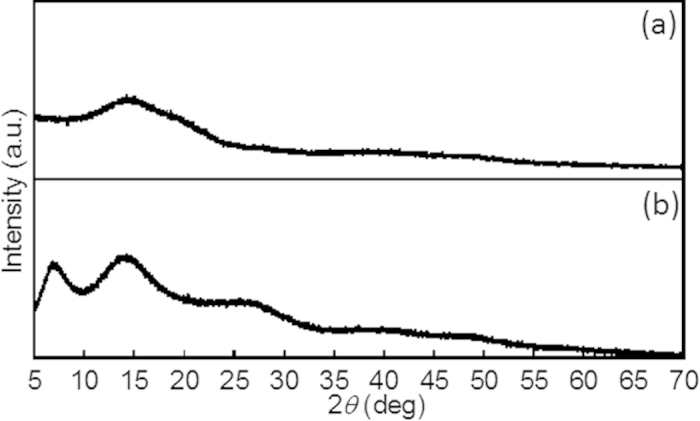
Synchrotron XRPD patterns obtained from **a**) SiO_2_ and **b**) SiO_2_/Eu nanoparticles (*λ* = 0.99933 Å).

Based on the synchrotron X-ray powder diffraction (XRPD) patterns in Fig. 3a, the SiO_2_ nanoparticles had an amorphous structure with a broad peak at approximately 15° 27, with an additional diffraction peak at 7.1° observed in the SiO_2_/Eu pattern (Fig. 3b). The additional peak indicates the formation of Eu crystal shells around the SiO_2_ nanoparticles. The crystal size, *D*, of the Eu shells was estimated using the Scherrer equation, *D* = 0.9*λ* / *β*cos*θ*  28, where *λ* is the X-ray wavelength and *β* is the full width in radians at half-maximum (FWHM) of the diffraction peak at the Bragg angle of *θ*. The Eu nanocrystal shell on the SiO_2_ nanoparticles was determined to be 1.3 nm in size, which is consistent with the SEM image. The nanostructure of the SiO_2_/Eu particles was evidently not affected by the coordination with phen, as determined from XRPD analysis and SEM images (Figs. S3 and S4).

**Figure 4:**
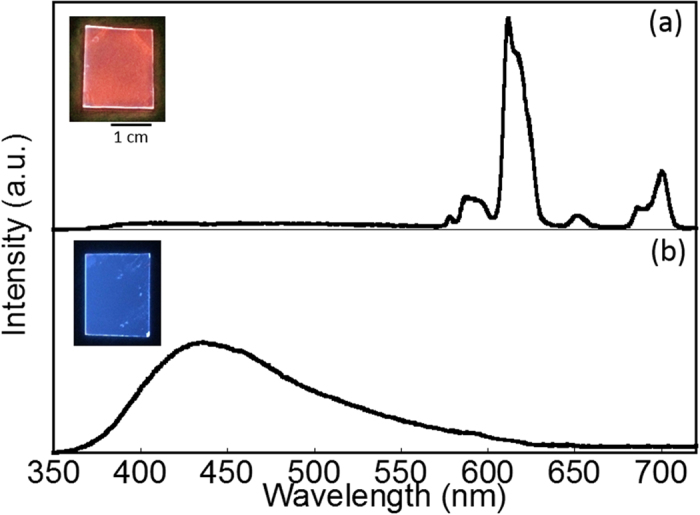
Luminescence spectra of **a**) as-prepared and **b**) sintered SiO_2_/Eu/phen nanoparticles (*λ*_ex_ = 280 nm). Insets show photographic images of each sample on glass substrates under UV irradiation. 385 nm band of a) is due to a tail of the ligand-centered emission of phen moiety filtered by a UV cut filter.

**Figure 5:**
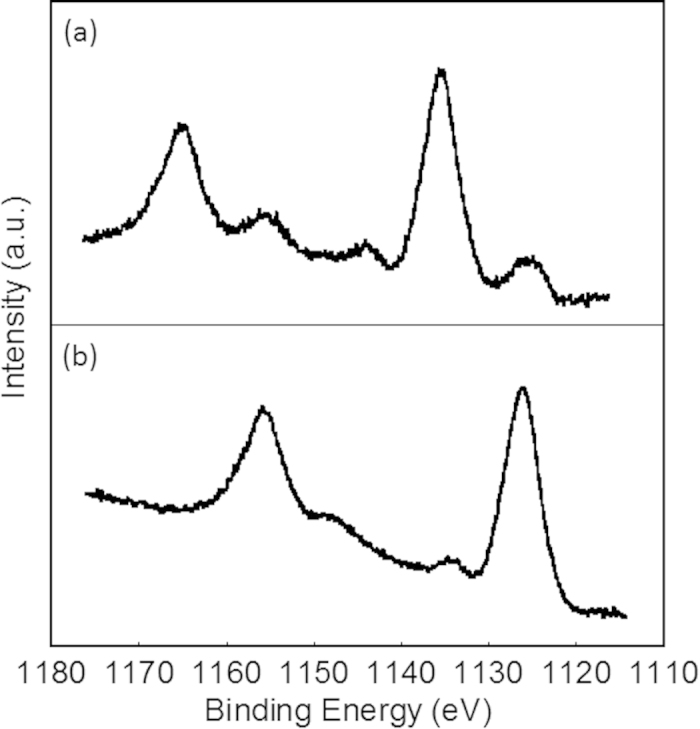
Eu3d XPS bands of **a**) as-prepared and **b**) sintered SiO_2_/Eu/phen nanoparticles.

Fig. 4 presents the luminescence spectra of SiO_2_/Eu/phen nanoparticles. Under UV light, the nanoparticles generated a bright red emission originating from the ff transitions of Eu^III^. Since the SiO_2_/Eu without phen cannot emit in any wavelength regions, this red emission may occur through energy transfer from the phen to the Eu^III^ at the interface of the SiO_2_/Eu nanoparticles. Interestingly, the emission colour of the SiO_2_/Eu/phen nanoparticles could be tuned from red to blue by sintering at 200 °C; after sintering for 60 min, the emission colour was completely changed to blue. The as-prepared SiO_2_/Eu/phen exhibited sharp emission bands at 578.2, 589.5, 611.1, 651.1 and 700.5 nm, assigned to the ^5^D_0_ → ^7^F_0_, ^5^D_0_ → ^7^F_1_, ^5^D_0_ → ^7^F_2_, ^5^D_0_ → ^7^F_3_ and ^5^D_0_ → ^7^F_4_ transitions of Eu^III^, respectively. Excitation spectra monitored at the ff emission band position correspond to the ππ* transition of phen (Fig. S5), confirming that energy transfer from the phen to the Eu^III^ was occurring within the nanoparticles. The red emission band was decreased following sintering at 200 °C and replaced by a broad blue emission band at approximately 434 nm (Fig. S6). This broad emission was the result of the allowed electric dipole 4f^6^5d → 4f^7^ transition of Eu^II^, via an allowed transition of Eu^II^ rather than energy transfer from the phen. The Eu3d XPS bands in Fig. 5 provided evidence for the reduction of Eu^III^ to Eu^II^ at the interface between the SiO_2_ and the phen. Eu3d XPS bands corresponding to Eu^III^ were observed at 1135.4 and 1165.2 eV in the case of the as-prepared SiO_2_/Eu/phen nanoparticles, while the sintered nanoparticles generated corresponding bands at lower energies (1126.2 and 1155.8 eV), assigned to Eu^II^ 29,30.

It is noteworthy that the interfacial SiO_2_/Eu/phen structure allowed the blue emissive Eu^II^ to be prepared in air and at the relatively low temperature of 200 °C. The morphology and thermal stability of the nanoparticles did not change before and after the sintering process, as shown in TGA and SEM images (Figs. S7 and S8). This transition to Eu^II^ was also stable; the blue emission properties of the material were maintained for more than three months. This reduction phenomenon of Eu was not observed in SiO_2_/Eu nanoparticles without phen and a pure Eu complex with phen. The reduction of Eu^III^ to Eu^II^ in a specially prepared matrix with a rigid inorganic crystal structure following high temperature treatment in air has been reported, and has been explained by a charge compensation model^31^,^32^,^33^. In the present reduction system, Eu ions were present at the interfaces between SiO_2_ nanoparticles and phen ligands, and the SiO_2_ surface acted as a rigid host while the phen functioned as an electron donor through coordination bonds. Thus, the reduced Eu^II^ state can be formed and protected from reaction with oxygen by the surrounding SiO_2_ and phen. To the best of our knowledge, this is the first reported fabrication of a stable Eu^II^-based emissive material using mild conditions (reaction in air and at low temperature) and an organic-inorganic hybrid nanostructure.

To quantitatively assess the effect of phen on the reduction of Eu ions at the interface, the absolute luminescence quantum yields, *ϕ*_ff_, and lifetimes, *τ*_ff_, of the SiO_2_/Eu/phen nanoparticles were estimated. The as-prepared SiO_2_/Eu/phen generated a Eu^III^ ff emission with *ϕ*_ff_ = 5.3% and *τ*_ff_ = 486.3 μs (Fig. S9). The total emission quantum yield of Eu^III^ sensitized by the ligand phen (*ϕ*_ff_) was determined by the triplet yield of the ligand (*ϕ*_ISC_), the efficiency of energy transfer (*η*_EnT_) and the efficiency of the metal centred luminescence (*η*_Ln_), as follows^34^.


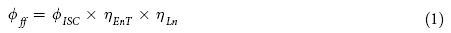


Because of the nπ* character of the ligand and the high spin-orbit coupling constants of the lanthanide ion, it can be assumed that *ϕ*_ISC_ was approximately 1 35,36. The value of *η*_Ln_ can be calculated from the observed emission lifetime (*τ*_ff_) and the radiative rate constant (*k*_R_) of the lanthanide ion, as shown below.


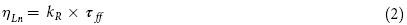


The *k*_R_ value of the emissive excited state, ^5^D_0_, is the sum of the spontaneous emission probabilities, *A*(0, *J*), to the lower ^7^F_*J*_ levels in Eu^III^, and can in turn be calculated from the following equation.


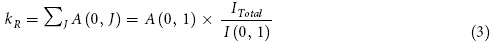


Here, *I*_Total_/*I*(0, 1) is the ratio of the total integrated intensity of the corrected Eu^III^ emission spectrum to the intensity of the ^5^D_0_ → ^7^F_1_ band. In this case, we obtained a value of 6.21 for *I*_Total_/*I*(0, 1). The spontaneous emission probability of the magnetic dipole ^5^D_0_ → ^7^F_1_ transition, *A*(0, 1), is virtually independent of the ligand field or the environment of the ions, and can be determined directly from the theoretically calculated dipole strength as follows.


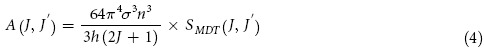


Here, *σ* is the energy gap between the excited (^5^D_0_) and the final (^7^F_1_) states (*σ* = 16963 cm^−1^), *n* is the refractive index (1.5 for the solid state metal-organic complex)^37^ and *S*_MDT_ (*J*, *J’*) is the magnetic dipole strength^38^. The latter parameter has been calculated theoretically for the ^5^D_0_ → ^7^F_1_ transition of Eu^III^ and found to have a value of 884 × 10^−8^ Debye^2^,^39^ leading to 45.7 s^−1^ for *A*(0, 1).

The calculated values for *k*_R_ and *η*_Ln_ (from Equations 2 and 3) obtained using the experimentally determined values of *τ*_ff_ and *I*_Total_/*I*(0, 1) were 283.2 s^−1^ and 0.384, respectively. The *k*_R_ value was less than that of a pure complex with phen (*ex.* 569.6 s^−1^ in [Eu(phen)_2_(NO_3_)_3_], Fig. S10 and Table S1), indicating that a more highly symmetrical environment was present in the vicinity of the Eu^III^ ions in the SiO_2_/Eu nanoparticles. These highly symmetrical conditions allow the Eu ion to function as a stable inorganic emissive compound. Thus, this organic-inorganic hybrid material simultaneously exhibits the photochemical and structural properties of both organic and inorganic materials, which may be responsible for the unusual reduction of Eu^III^ to Eu^II^ in air at a low temperature. From Equation 1, the value of the energy transfer efficiency, *η*_EnT_, from phen to Eu^III^ on the SiO_2_/Eu nanoparticles is estimated to be 0.385. In contrast, the value of *η*_EnT_ is almost 1 in the solid state molecular structure [Eu(phen)_2_(NO_3_)_3_]. The considerably lower *η*_EnT_ of the SiO_2_/Eu/phen nanostructure suggests the existence of an alternative energy migration pathway, such as a ligand (phen) to metal (Eu^III^) charge transfer (LMCT)^40^,^41^,^42^,^43^. The presence of an LMCT pathway indicates the ability of the phen to donate electrons to Eu ions, which may result in the unusual reduction of Eu^III^ to Eu^II^ through a thermally activated process. In this reduction-induced emission system, SiO_2_ nanoparticles as host materials is supposed to keep the valence of Eu^II^ at the interface.

**Figure 6:**
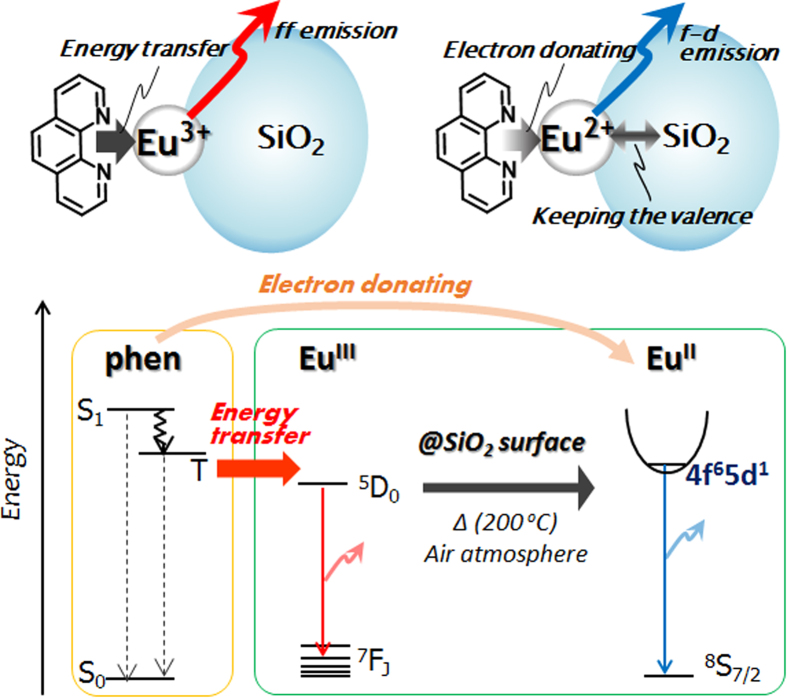
Energy diagram for as-prepared and sintered SiO_2_/Eu/phen nanoparticles.

After sintering at 200 °C, a blue emission band due to the Eu^II^ appeared around 435 nm with *τ* = 11.59 μs (Fig. S9). The *ϕ* of the Eu^II^ in the SiO_2_/Eu/phen eventually reached a value of 7.2%, higher than that of the Eu^III^ state because of the allowed electric dipole 4f^6^5d → 4f^7^ transition. Following sintering at 200 °C, Eu^III^ emission was no longer observed. The surfaces of the SiO_2_/Eu particles were less likely to be attacked by oxygen because of the coordination with phen, and therefore the Eu^II^ state in the SiO_2_/Eu/phen was very stable and the ions were prevented from being re-oxidized, even in air. The reduction-induced emission phenomena are summarized by the energy diagram in Fig. 6.

## Conclusions

In conclusion, we discovered a novel emission phenomenon associated with the reduction process in an interfacial complex formed on inorganic nanoparticles. In this study, a Eu-coated SiO_2_ nanostructure was developed, consisting of an interfacial complex of Eu and phen at the solid surfaces. The as-prepared SiO_2_/Eu/phen nanoparticles exhibited sharp red emission via energy transfer from the phen to the Eu^III^. After sintering at 200 °C in air, the emission was tuned from red to blue. The blue emission resulted from Eu^II^, indicating that the unusual reduction of Eu^III^ to Eu^II^ under mild conditions was successfully accomplished for the first time. The existence of two different stable oxidation states with characteristic emissions, blue emissive Eu^II^ and red emissive Eu^III^, suggests significant potential applications as novel luminescent materials with inorganic-organic hybrid structures. For instance, our colour-tunable SiO_2_ nanoparticles with Eu ions, as having less toxicity, will be greatly beneficial for biological and biomedical applications. Additionally, this redox-active interface between inorganic and organic materials may provide a new photon energy conversion system such as artificial photosyntheses and solar cells. Studies are now underway to fabricate a photoelectron conversion system based on the Eu interfacial complex by way of using a metal oxide with an appropriate redox potential, such as mesoscopic TiO_2_.

## Methods

### Sample preparation

SiO_2_/Eu nanoparticles were prepared by the sol-gel method. SiO_2_ nanoparticles (QS-20, Tokuyama Co.) were suspended in ethanol (10 wt%) followed by the addition of a 50 mM ethanol solution of EuCl_3_ (Kanto Chemicals Co., Inc.) at 70 °C for 30 min. The resulting colloidal suspension containing SiO_2_ and Eu ions was dropped onto a quartz substrate that had been sequentially cleaned ultrasonically in acetone, isopropanol and ultra-pure water (10 min in each solvent). Following treatment at 110 °C for 15 min, SiO_2_ nanoparticles coated with Eu^III^ oxides or hydroxides were obtained. To generate complexation at the particle surfaces, the glass substrate holding the nanoparticles was immersed in a 1 mM ethanol solution of phen (Kanto Chemicals Co., Inc.) at 75 °C for 60 min. After drying, the nanoparticles were further sintered at 200 °C in air.

### Apparatus

SEM images were obtained on a ZEISS ULTRA 55 microscope equipped with a secondary in-lens electron detector, together with a Bruker-QUANTAX detector for EDS studies. X-ray photoelectron spectroscopy (XPS) was performed using a KRATOS AXIS ULTRA DLD equipped with a monochromatic Al-Kα X-ray source (1253.6 eV); the binding energies were calibrated at the Au 4f level (84.0 eV). Synchrotron X-ray powder diffraction (XRPD) patterns were obtained with a large Debye-Scherrer camera installed at the SPring-8 BL02B2 beamline, using an imaging plate as the detector^44^ and an incident X-ray wavelength of 0.99933 Å. Luminescence spectra were recorded on a Horiba Jobin-Ybon Fluorolog 3–22 with a UV cut filter. The emission decay curves were acquired using a Quantaurus-Τau C11367-12 (Hamamatsu Photonics K. K.) with excitation via a xenon flash lamp with a band-path filter (*λ*_ex_ = 280 nm). Fluorescence quantum yields were measured by using a C9920-02 Absolute PL Quantum Yield Measurement System (Hamamatsu Photonics K. K.)^45^,^46^,^47^.

## Additional Information

**How to cite this article**: Ishii, A. and Hasegawa, M. An Interfacial Europium Complex on SiO_2_ Nanoparticles: Reduction-Induced Blue Emission System. *Sci. Rep.* **5**, 11714; doi: 10.1038/srep11714 (2015).

## Supplementary Material

Supplementary Information

## Figures and Tables

**Figure 1:**
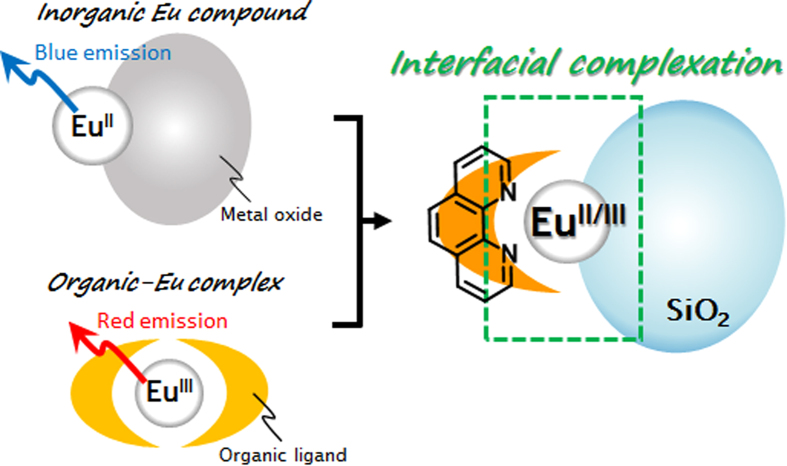
Schematic illustration of SiO_2_ nanoparticles coated with Eu ions followed by complexation with phen at the interface.
