# Supplementary material for: An Interfacial Europium Complex on SiO2 Nanoparticles: Reduction-Induced Blue Emission System
Source: Sci Rep. 2015 Jun 30;5:11714. doi: 10.1038/srep11714 (PMC4485170; doi:10.1038/srep11714)
Supplement: Supplementary Information [file srep11714-s1.doc]

*Supplementary information*

**An Interfacial Europium Complex on SiO_2_ Nanoparticles:**

**Reduction-Induced Blue Emission System**

Ayumi Ishii, and Miki Hasegawa

College of Science and Engineering, Aoyama Gakuin University, 5-10-1 Fuchinobe, Chuo-ku, Sagamihara, Kanagawa, 252-5258, Japan

E-mail: ayumi@chem.aoyama.ac.jp (A.I.); hasemiki@chem.aoyama.ac.jp (M.H.)

**Figure S1.** N1s XPS bands of phen coordinated with SiO_2_/Eu nanoparticles, compared with those of phen (dotted line) in the solid state.

**Figure S2.** a) Low and b) high magnification SEM images of SiO_2_ nanoparticles.

**Figure S3.** Synchrotron XRPD pattern obtained from SiO_2_/Eu/phen nanoparticles (*λ* = 0.99933 Å).

**Figure S4.** SEM image of SiO_2_/Eu/phen nanoparticles.

**Figure S5.** Excitation spectra of as-prepared (red) and sintered (blue) SiO_2_/Eu/phen nanoparticles monitored at a) 611 and b) 434 nm.

**Figure S6.** Luminescence spectra of SiO_2_/Eu/phen nanoparticles (*λ*_ex_ = 280 nm) after sintering for a) 10 and b) 30 min.

**Figure S7.** TGA curves of as-prepared (red) and sintered (blue) SiO_2_/Eu/phen nanoparticles.

**Figure S8.** Low magnification SEM images of a) as-prepared and b) sintered SiO_2_/Eu/phen nanoparticles.

**Figure S9.** Luminescence decay curves of a) as-prepared and b) sintered SiO_2_/Eu/phen nanoparticles monitored at a) 611 and b) 434 nm (*λ*_ex_ = 280 nm).

**Figure S10.** Luminescence spectrum of [Eu(phen)_2_(NO_3_)_3_] in the solid state (*λ*_ex_ = 280 nm).

**Table S1.** Luminescence lifetime *τ*_ff_, absolute luminescence quantum yield *ϕ*_ff_ and the ratio of the emission intensity *I_Total_*/*I*(0, 1) values for [Eu(phen)_2_(NO_3_)_3_] in the solid state (*λ*_ex_ = 280 nm).

| *τ*_ff_ (μs) | *ϕ*_ff_ | *I*_total_/*I*(0,1) |
| --- | --- | --- |
| 1276 | 0.79 | 12.7 |
